# Real-Time Vehicle-Detection Method in Bird-View Unmanned-Aerial-Vehicle Imagery

**DOI:** 10.3390/s19183958

**Published:** 2019-09-13

**Authors:** Seongkyun Han, Jisang Yoo, Soonchul Kwon

**Affiliations:** 1 Department of Electrical Engineering, Kwangwoon University, 20 Kwangwoon-ro, Nowon-gu, Seoul 01897, Korea; skhan3410@naver.com (S.H.); jsyoo@kw.ac.kr (J.Y.); 2 Department of Smart Convergence, Kwangwoon University, 20 Kwangwoon-ro, Nowon-gu, Seoul 01897, Korea

**Keywords:** vehicle detection, object detection, UAV imagery, convolutional neural network

## Abstract

Vehicle detection is an important research area that provides background information for the diversity of unmanned-aerial-vehicle (UAV) applications. In this paper, we propose a vehicle-detection method using a convolutional-neural-network (CNN)-based object detector. We design our method, DRFBNet300, with a Deeper Receptive Field Block (DRFB) module that enhances the expressiveness of feature maps to detect small objects in the UAV imagery. We also propose the UAV-cars dataset that includes the composition and angular distortion of vehicles in UAV imagery to train our DRFBNet300. Lastly, we propose a Split Image Processing (SIP) method to improve the accuracy of the detection model. Our DRFBNet300 achieves 21 mAP with 45 FPS in the MS COCO metric, which is the highest score compared to other lightweight single-stage methods running in real time. In addition, DRFBNet300, trained on the UAV-cars dataset, obtains the highest AP score at altitudes of 20–50 m. The gap of accuracy improvement by applying the SIP method became larger when the altitude increases. The DRFBNet300 trained on the UAV-cars dataset with SIP method operates at 33 FPS, enabling real-time vehicle detection.

## 1. Introduction

In recent years, studies have been conducted to apply a large amount of information obtained from Unmanned Aerial Vehicles (UAVs) to various systems. Representative UAV applications exist in social-safety, surveillance, military, and traffic systems, and the field is increasingly expanding [1,2,3,4,5,6,7]. In this application, vehicle detection, which is detecting the position and size of vehicles in UAV imagery, is very important as background information. Zhu et al. [5] and Ke et al. [6] proposed a vehicle-flow- and density-calculating system in UAV imagery using the vehicle-detection model. Yang et al. [7] proposed an Intelligent Transportation System (ITS).

Traditional methods are less accurate because of poor generalization performance, and only vehicles on asphalt roads are detectable in top-view images, where only relatively standardized vehicle shapes are shown [8,9,10]. However, AlexNet [11] won the 2012 ImageNet Large Scale Visual Recognition Competition (ILSVRC) [12] and showed excellent generalization performance on a Convolutional Neural Network (CNN). As a result, in 2015, a CNN was able to classify more accurately than humans [12]. Various CNN-based object-detection models have been proposed, such as the Single Shot MultiBox Detector (SSD) series [13,14,15] and Region proposals with CNNs (RCNN) series [16,17,18], which utilize CNN. A diversity of vehicle-detection methods has been proposed using various CNN-based object detectors, but these UAV imagery vehicle-detection methods fail to find small objects and operate at low altitudes [19] using YOLOv2 [20]. There is also a real-time problem using complex models to improve accuracy [4,5,21,22], like Faster-RCNN [18], deep and complex SSD [13], and YOLOs [15,20].

The MS COCO [23] and PASCAL VOC [24] used in the training of a general object-detection model consists of front-view images. In addition, each dataset has labels that are not needed for UAV imagery vehicle detection, such as suitcase, fork, wine glass or bottle, potted plant, and sofa, respectively. Vehicles in UAV imagery captured at a high altitude have different composition and distortion peculiarities than general front-view images. For those reasons, vehicle detection using a model trained with a general object detection dataset is not accurate in UAV imagery.

Therefore, in this paper, we propose a real-time vehicle-detection method in bird-view UAV imagery using a lightweight single-stage CNN-based object detector. First, we designed a DRFB module and DRFBNet300, which is a light and fast detection model that uses the MobileNet v1 backbone [25]. Our DRFB module has multi-Receptive Field-size branches to improve the expressive power of feature maps, using dilated convolution [26] to minimize increments of computational complexity. We propose the UAV-cars dataset, which includes distortion peculiarities of UAV imagery to train object-detection models. We also propose a Split Image Processing (SIP) method to improve the accuracy of the detection model. Our SIP method improves accuracy by using divided input frames, different from existing CNN-based object-detection methods. Thus, using the DRFBNet300 trained on the UAV-cars dataset and by using the SIP method, we propose a real-time bird-view UAV imagery vehicle-detection method.

In Section 3.1, we describe DRFBNet300 using the DRFB module, which is optimized for small-object detection. Section 3.2 outlines the SIP method, which improved the accuracy of the object-detection model. In Section 3.3, we describe the UAV-cars dataset that contains the distortion peculiarities of vehicles in UAV imagery. Section 3.4 describes the overall flow of our vehicle-detection method. In Section 4, we lay out the environment of the experiments. Section 5.1 outlines a performance comparison between each model using the MS COCO dataset. In Section 5.2, we compare the performance of the models trained on our UAV-cars dataset with and without the SIP method.

All models used MobileNet v1 [25] with one *Width Multiplier* (α) and *Resolution Multiplier* (ρ) as a backbone network for fast detection. In the MS COCO experiment, SSD300 [13], RFBNet300 [14], YOLOv3 320 [27], FSSD300 [28], and our DRFBNet300, which are single-stage object detectors, were used for the performance comparison. In the UAV-cars dataset experiment, SSD300, RFBNet300, and our DRFBNet300, which are lightweight single-stage methods, were used for the comparison. At each model, the number means input size.

## 2. Related Work

Vehicle-detection algorithms require a high-end computing model that needs high amounts of power. It is difficult to mount these high-end computing models directly on small battery-powered UAVs. Therefore, most of them use precaptured or transmitted images from the UAV and run detection algorithms on the server PC [1,2,3,4,5,6,7,8,9,10,19,21,22,29,30,31,32,33,34].

Vehicle detection has been widely studied as a background research area for various applications, like surveillance systems and traffic systems [1,2,3,4,5,6,7]. Various studies using traditional handcrafted methods have been carried out. Zhao et al. [8] detected vehicles using the edge-contour information of vehicles on the road in top-view low-resolution images. Breckon et al. [29] detected vehicles using a cascaded Haar classifier [35] in bird-view UAV imagery. Shao et al. [31] found vehicles using various algorithms, such as Histogram of Oriented Gradients (HOG) [36], Local Binary Pattern (LBP) [37], and exhaustive search in top-view high-resolution images. Yang et al. [9] tracked vehicles with Scale Invariant Feature Transform (SIFT) [38] and the Kanade–Lucas–Tomasi (KLT) feature tracker [39] after finding a vehicle using blob information in top-view images. Xu et al. [10] improved the original Viola–Jones object-detection method [35] to enhance the accuracy of UAV imagery vehicle-detection models at low altitudes. However, these handcrafted methods are not robust, and are only accurate in certain environments, such as on roads with one direction. They are also only optimized for top-view images, where vehicles are seen as a formulaic square shape, or for low-altitude UAV imagery.

To overcome the low generalization performance of traditional handcrafted methods, various UAV imagery vehicle-detection methods using CNN-based object detectors have been proposed. Yang et al. [6] proposed the Enhanced-SSD, which modified the SSD structure [13], and detect vehicles in ultrahigh-resolution UAV imagery. Tang et al. [33] proposed a UAV imagery vehicle-detection model using the original structure of YOLOv2 [20]. Radovic et al. [19] used the structure of YOLO [15] to detect vehicles in UAV imagery. Xu et al. [22] proposed a deeper YOLO model, DOLO, using the structure of YOLOv2. Xie et al. [34] proposed a UAV imagery vehicle-detection model by modifying the structure of RefineDet [40]. Fan et al. [21] proposed a vehicle-detection model using Faster-RCNN [18], which is a two-stage method. However, most of these methods use simple top-view images. They also designed the models to operate at low altitudes [19] or to use ultrahigh-resolution images [6], and heavyweight models are used to achieve high accuracy, which has high computational complexity [6,21,22,33,34].

## 3. Proposed Method

**Figure 1:**
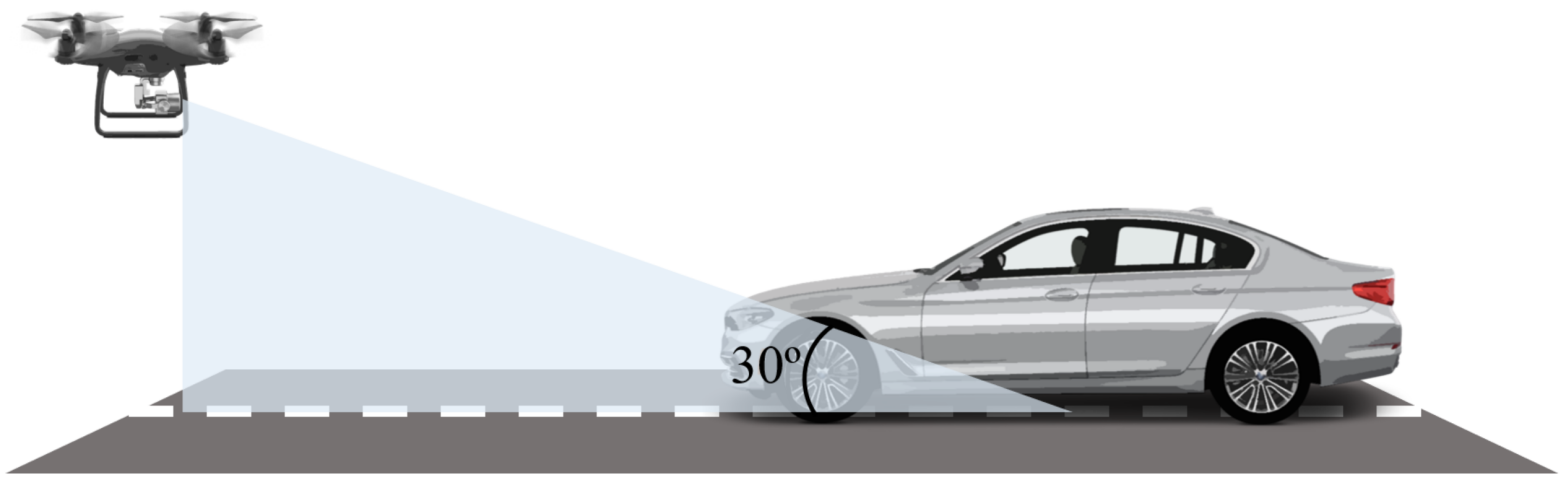
Unmanned-aerial-vehicle (UAV) imagery-capturing schematic concept.

Figure 1 shows imagery capturing the schematic concept of the proposed vehicle-detection method in bird-view UAV imagery. The angle of the camera was 30 degrees from the ground, and the video was taken at various altitudes while maintaining the camera angle. The altitude of the UAV was 10–50 m above ground. The vehicle-detection model used a prerecorded bird-view UAV image to infer the location and size of the vehicle on the server PC. Imaging was done using a built-in UAV camera; its detailed specifications are covered in Section 4.1, and those of the server PC used in the experiment are covered in Section 4.2.

**Figure 2:**
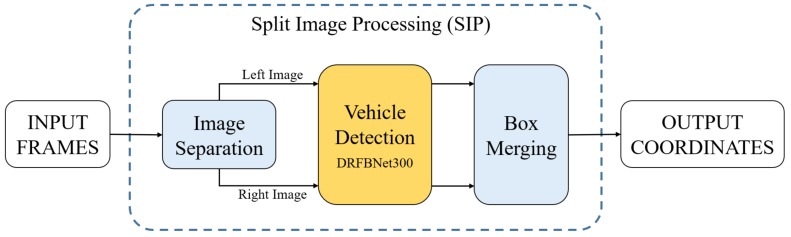
Overall flowchart of our UAV imagery vehicle-detection method.

Figure 2 shows the overall flowchart of the proposed UAV imagery vehicle-detection method. First, the input image was separated left and right through the Image Separation part of the SIP method. The two separated images were inputted, respectively, to the DRFBNet300 trained on the UAV-cars dataset. Next, using the coordinates indicating the position and size of objects found in the overlapped area at each separated image, the overlapping results were combined at the Box Merging part. Finally, result boxes were drawn on the input image using the generated coordinate values. In Section 3.1, we explain the DRFB module and DRFBNet300, used for vehicle detection. Section 3.2 describes the SIP method that separates the input image of DRFBNet300 and combines or removes duplicated coordinates from the inference result. Section 3.3 describes the UAV-cars dataset, which is used to train and validate the proposed vehicle model. In Section 3.4, we discuss the overall framework.

### 3.1. DRFBNet300

Speed and precision, the main performance indices of the object detector are directly related to the structure of the backbone network and the meta-architecture of the detector. Therefore, there are performance differences according to the meta-architecture even for the same backbone network [41]. To improve the accuracy of the object-detection model, several studies use a heavyweight backbone network such as ResNet [42] or VGGs [43], or meta-architecture like the RCNN series [16,17,18]. Such a heavyweight structure is computationally complex and expensive, resulting in the real-time problem of the object-detection model. This problem can be solved using a lightweight backbone like MobileNet v1 [25] or single-stage meta-architecture such as SSD [13]. However, a lightweight structure results in low accuracy because of the lack of network capacity.

In this paper, we designed a DRFB module to improve the accuracy of MobileNet v1 backbone SSD300 [13], which is a light and fast detector. The values of *Width Multiplier* (α) and *Resolution Multiplier* (ρ) of MobileNet v1 were 1. The proposed DRFB module was designed based on the human population Receptive Field (pRF) [44], Inception series [45,46,47], and RFBNet [14]. The DRFB module improved the quality of feature maps with weak expressive power. DRFBNet300 is an object-detection model using our DRFB module and RFB basic module [14] on the MobileNet v1 backbone SSD300 structure.

DRFB module. Using a multisized Receptive Field (RF) branch structure rather than a fixed-sized RF layer in CNN increases scale invariance and produces better-quality feature maps [48,49]. In addition, if the Inception family-based module [45,46,47] that concatenates the feature maps generated by the multisized RF convolution is applied to the CNN, the expressiveness of the feature maps and the accuracy of the model are improved, with faster training speed [46,47]. These Inception module-based approaches have been verified in classification, semantic-segmentation, and object-detection tasks [14,47,48,49].

**Figure 3:**
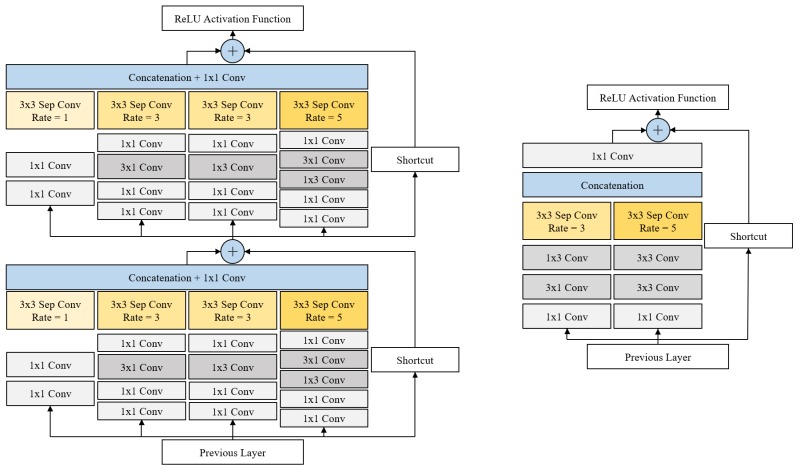
Structure of the (**left**) DRFB module and (**right**) RFB basic module.

The proposed DRFB (Deeper Receptive Field Block) module was connected to feature maps for detecting small objects and consists of branches with variously sized RFs. The left-hand side of Figure 3 shows the structure of our DRFB module. Each branch uses dilated convolution [26] to generate good-quality feature maps using large RF. The module has a shortcut branch of ResNet [42] and Inception-ResNet V2 [46], and follows the multibranch structure of inception [45,46]. This makes it possible to enhance the expressiveness of the feature maps and speed up model training while minimizing parameter increase.

Our DRFB module used 1×1 convolution to increase nonlinearity and depth. This minimizes the amount of computation increases and improves the capacity of the structure [50]. Instead of using 3×3 convolutions, 1×3 and 3×1 convolutions were used to reduce computational complexity with nonlinearity increments. The depth of the 5×5-dilated convolution branch was deeper than other branches. The SSD series object-detection model deduces the position, size, and label of multiple objects in a single-input image at once. Therefore, we used a deeper structure to increase the capacity of the large RF branch by adding nonlinearity in order to extract better features from objects that were scattered in the image. We also used a cascaded structure to enhance the expressiveness of the feature maps. The deeper branch had a bottleneck structure based on Szegedy et al. [45,46] to increase efficiency while minimizing the number of parameter increasing.

In each structure in Figure 3, each layer in every branch includes batch normalization and ReLU activation after the convolution layer (**Conv**). However, a separable convolution (**Sep Conv**), shortcut and the concatenation layer did not include an activation function. Table 1 shows the number of channels in each layer before the DRFB module was cascaded. In Table 1, the top and bottom row mean input and output, respectively, and each number sequentially indicates the number of input/output channels, the application of batch normalization (**BN**), and the ReLU activation function (**ReLU**). The DRFB module was composed of the structure cascade in Table 1. The spatial size of all inputs and outputs equaled 19×19. The shortcut branch, shown in Figure 3, was multiplied by a scale factor (0.1 [14]) and added to each feature map. The structure of the RFB basic module was the same as the one of Liu et al. [14].

DRFBNet300. The SSD object-detection model has various combined backbone versions. Among them, the MobileNet v1 backbone SSD300 uses depthwise convolution [25], which reduces the number of parameters and computational complexity, preserving its accuracy. However, the SSD object detector was trained to detect small-sized objects using feature maps from the front side of the feature extractor. Accordingly, feature maps for small-sized object detection have relatively low expressive power. Therefore, the SSD could quickly detect objects, but overall accuracy is low.

**Figure 4:**
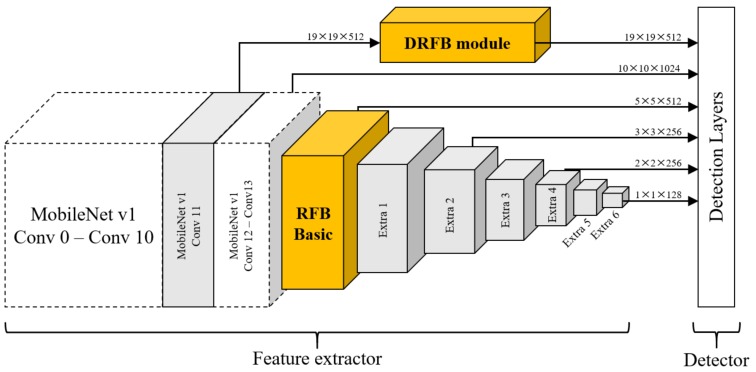
DRFBNet300 structure.

In this paper, we propose our DRFB module-applied MobileNet v1 backbone SSD300 with RFB basic module [14] and define it as DRFBNet300. The right-hand side of Figure 3 shows the structure of the RFB basic module. Figure 4 shows the structure of the proposed DRFBNet300. For the backbone network, we used ImageNet [51] pretrained MobileNet v1. All of the structures in Figure 4 were identical to MobileNet v1 backbone SSD300 except the RFB basic and DRFB modules. The feature extractor consisted of the MobileNet v1 backbone, DRFB module, RFB basic module, and six additional convolution layers. The quality of the feature maps for small-object detection, 19×19×512 shapes, was enhanced through the DRFB module. The RFB basic module was connected to the front side of the extra layers. As a result, the expressiveness of the feature maps for large-object detection was enhanced, improving the overall accuracy of the detection model.

### 3.2. Split Image Processing

In general object-detection methods, the input image of a single-stage detector is resized to a specific size. An SSD is divided into SSD300 and SSD512 according to the resized input image [13]. Single-stage object-detection models deduce the position, size, and label of the object in the input image with only one network forward pass. Therefore, the SSD512 uses high-resolution input detect objects relatively well, but the SSD300 does not. On the other hand, the SSD300 using small-sized inputs deduces results using only 9.7% (90,000 pixels) of the input image when the input size is 720P (921,600 pixels). This makes SSD300 relatively fast, but low accuracy is inevitable.

**Figure 5:**
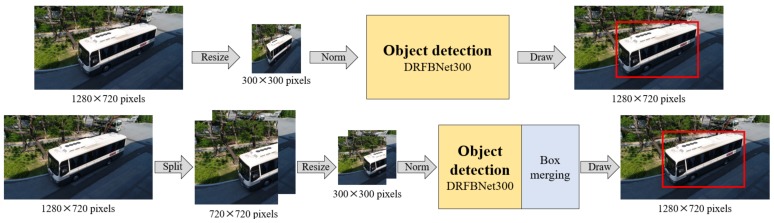
Schematic concept of (**top**) existing object-detection method and (**bottom**) proposed Split Image Processing (SIP) method.

Therefore, in this paper, we propose a SIP method that reduces information loss at the input-image-resizing process of the network. The bottom side of Figure 5 shows the schematic concept of the SIP method. Unlike the conventional method shown in the upper part of Figure 5, the detection method with SIP inputs separated images into two segments at the Image Separation part and outputs the final result through the Box Merging part. Overall flow is shown in Figure 2.

Image Separation. A single input image is separated into two images so that 12.5% of the original width is overlapped at the center. If the input image is 720P (1280×720 pixels), then 160×720 pixels are overlapped at the center. A single 720P image is separated into two 720×720 pixel images. The separated images are inputted in object-detection model DRFBNet300 through normalization. The network infers the positions of the objects in each left and right image. The Box Merging part of the SIP method merges the coordinates of the objects in the overlapped area to generate the final result.

**Figure 6:**
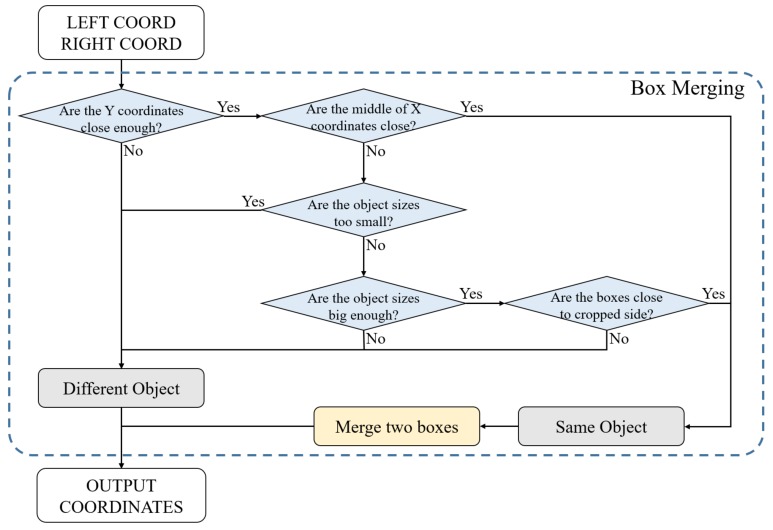
Box merging flowchart.

**Figure 7:**
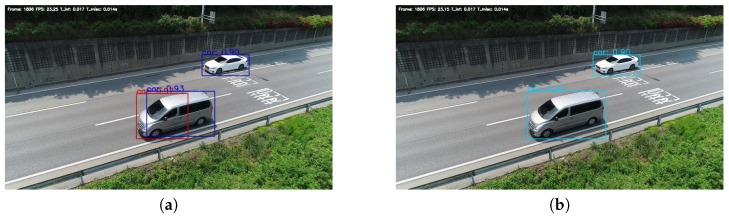
Experiment results (**a**) before and (**b**) after applying the Box Merging part.

Box Merging. Figure 6 shows a flowchart of the Box Merging part. All thresholds were 720P image referenced values, and optimal values were obtained through experiments. The object-detection model outputs a result in a coordinate format. Values used in the Box Merging part are the coordinate values of objects in the overlapped area. If the detector locates the same object in the overlapped area at each left and right image, the box is truncated or overlapped, as shown in the left image in Figure 7. This happens when objects in the overlapped area are simultaneously detected in the left and right images.

In general, when the same object is detected in each of the left and right UAV imagery, the difference of the *Y* coordinates is not large. Using this, considering the difference between minimum (top) and maximum (bottom) *Y* values between each left and right box, if the difference is larger than 20 pixels, it is decided as another object. In the comparison of *Y* coordinates, the top and bottom values are separately compared in the overlapping boxes. The final result can be true when both respective values satisfy the condition. When the *Y* coordinate-value condition is satisfied, the Box Merging part uses the center point between the same objects of the result. To do this, the *X*-coordinate center point of each box was calculated, and, if the distance between them was less than 40 pixels, it was decided as the same object. If the size of the bounding box was smaller than 30×30 pixels even if all of the previous conditions were satisfied, it was decided as another box. This is a condition that considers when the size of the vehicles is very small at a high altitude. If the box size was larger than 160×160 pixels, the maximum *X* value of the left image box was in the range of 710–720 pixels, and if the minimum *X* value of the right image box was in the range of 560–570 pixels, it was decided to the same object. This is a condition that considers when the size of the vehicles is very large at low altitude. Figure 7 shows the results before and after applying the Box Merging part.

### 3.3. UAV-Cars Dataset

**Figure 8:**
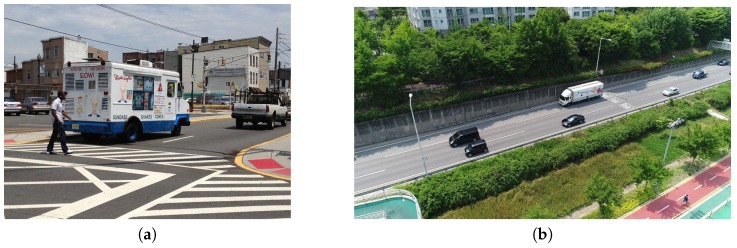
Vehicle-feature difference between (**a**) MS COCO and (**b**) UAV imagery.

To train the general object-detection model, most studies used datasets such as MS COCO [23] or PASCAL VOC [13,14,15,16,17,18,20,24,40,50]. Each dataset has 81 and 21 labels, including backgrounds, and labels such as frisbee, hot dog, and potted plant. These labels are very insignificant in UAV imagery vehicle detection. Furthermore, UAV imagery is captured using a wide-angle camera, resulting in object composition, ratio, and angle distortion. Most general object-detection datasets consist of front-view images, and even equally labeled objects have different characteristics. Figure 8 shows feature differences of the vehicle between MS COCO and UAV imagery. If the general object-detection dataset is used for UAV imagery vehicle-detection model training, detection accuracy deteriorates because it does not have the peculiarities of UAV imagery.

In this paper, we propose a dataset for vehicle detection in bird-view UAV imagery and UAV-cars. The UAV-cars dataset includes a training and a validation set. The training set consists of 4407 images containing 18,945 objects, and the validation set consists of 628 images containing 2637 objects. To generate the dataset, the vehicles on the road were directly captured using the built-in UAV camera, and then the video was sampled to make images at a constant frame rate. A total of 5035 images were used to generate ground truth (GT) using LabelImg [52]. We used LabelImg to create the GT boxes and save coordinates in the form of XML files. In addition, 628 images that were not included in the training set were used as the validation set. The validation set included the altitude condition, which was 10 m intervals from 10 to 50 m.

We used a camera equipped with a wide-angle lens and UAV to capture the UAV imageries. Detailed specifications of UAV and camera will be covered in Section 4.1. All images were taken in various vehicle compositions with altitude changes between 10 and 50 m. As a result, the UAV-cars dataset contained all various distortion peculiarities of UAV imagery.

### 3.4. Vehicle Detection in UAV Imagery

Network Training. We used GPU-enabled Pytorch 1.0.1, which is the deep learning library, to implement DRFBNet300. Our training strategies were similar to SSD, including data augmentation and the aspect ratios of the default box. The size of the default box was modified to detect small objects well. For weight-parameter initialization, the weight values of ImageNet [51]-pretrained MobileNet v1 were used for the backbone network. All remaining layers were initialized using the MSRA method [53]. The loss function used in the training phase was multibox loss [13]. The Stochastic Gradient Descent (SGD) momentum optimizer was used to optimize the loss function. DRFBNet300 was trained on the UAV-cars training set for 150 epochs. Further details are covered in Section 4.3.

Vehicle detection. The proposed vehicle-detection model in bird-view UAV imagery is implemented by applying the SIP method to DRFBNet300 trained on the UAV-cars dataset. Figure 2 shows a flowchart of the entire vehicle-detection method. We use precaptured bird-view UAV imagery as input. The video input to the program was divided into frames and conveyanced to our vehicle-detection model. The input frame was separated into left and right images through the Image Separation part. The separated images were fed to DRFBNet300 pretrained on the UAV-cars dataset to infer the coordinates and scores. The Box Merging part used the coordinates of the bounding boxes inferred from DRFBNet300 to merge redundant detection results when objects were in the overlapped area. Finally, the completed coordinate values were drawn in the bounding-box shape on the input image, and results were displayed on the screen and saved.

## 4. Experimental Environment

### 4.1. UAV Specification

**Figure 9:**
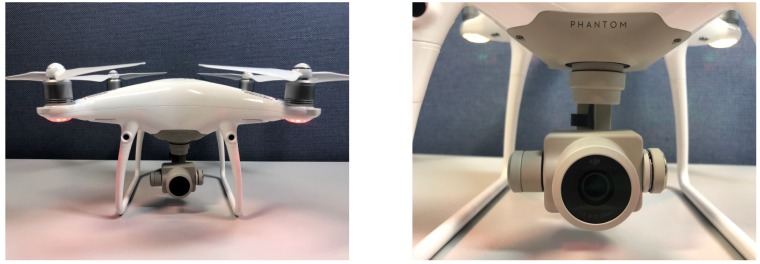
DJI Phantom 4 Advanced UAV (**left**) and its built-in camera (**right**).

The experiment used images taken with the DJI Phantom 4 Advanced model (Shenzhen, China). The weight of the fuselage was 1368 g and the size was diagonally 350 mm except for the propellers. The fuselage was equipped with four front- and bottom-side cameras, a GPS, and a gyroscope for the autonomous flight system. The UAV used these sensors to fly at a vertical error of ±10 cm. The built-in camera used a 20M pixel one-inch CMOS sensor and it was equipped with an 8.8/24 mm lens of 84 FOV. The gimbal that connects the camera to the fuselage has three axes to compensate for yaw, pitch and roll motion. All images were shot at 720P (1280×720 pixels) with 30 FPS. Figure 9 shows the UAV and its built-in camera used in the experiment.

### 4.2. Experiment Environment

During the implementation of the proposed method, we used GPU-enabled Pytorch 1.0.1. Pytorch uses CUDA 9.0 and the cuDNN v 7.5 GPU library. Table 2 shows the specifications of the server PC used for model training and operating our vehicle-detection model.

### 4.3. Training Strategies

The same training strategies were applied to training models using MS COCO [23] and the UAV-cars dataset. Data augmentation, including distortions such as cropping, expanding, and mirroring, was applied to the training phase. Data normalization was applied for fast training and global-minima optimization using mean RGB values (104,117,124) of MS COCO [14]. The models were trained with 32 batch sizes during 150 epochs. The learning rate started at $2\times 10^{-3}$ and was reduced by 1/10 at 90, 120, and 140 epochs, respectively. We applied a warm-up epoch [54] that helped global-minima convergence during the initial five epochs, linearly increasing the learning rate from $1\times 10^{-6}$ to $2\times 10^{-3}$ during the very first five epochs. The SGD momentum using a 0.9 momentum coefficient and $5\times 10^{-4}$ weight decay coefficient was used as an optimizer.

Different methods were applied to the backbone and the remaining layers to initialize the weight parameters of the network. The initial weight parameter of the backbone network used ImageNet [51] pretrained MobileNet v1 [25], and all other layers were initialized using the MSRA method [53].

## 5. Experimental Results

### 5.1. MS COCO

In this experiment, we used the MobileNet v1 [25] backbone SSD300 [13], RFBNet300 [14], YOLOv3 320 [27], FSSD300 [28] and our DRFBNet300, which are single-stage object-detection methods. We trained each model using MS COCO *trainval35k* [23]. The training strategies in Section 4.3 were used for each model. All models in this experiment were evaluated using MS COCO *minival2014* [23].

**Figure 10:**
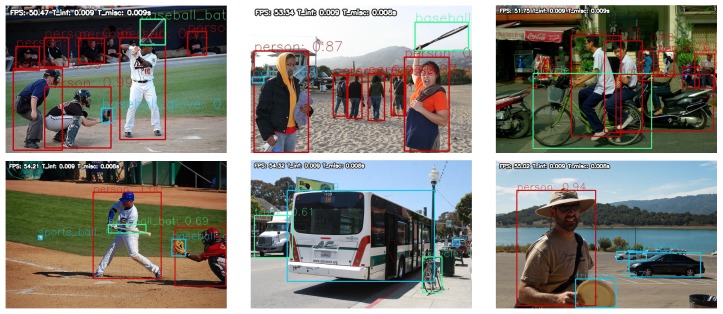
Object-detection results of DRFBNet300 on MS COCO val2017.

Table 3 shows the speed and mean Average Precision (mAP) of each model trained on MS COCO. The experiment result showed that the proposed DRFBNet300 achieves 21 mAP. This is the highest mAP score compared to the SSD300 and RFBNet300, which are lightweight single-stage object-detection models running in real time. The network inference of DRFBNet300 also only took 22.3 ms, meaning real-time detection is possible at about 45 FPS. The FSSD300 and YOLOv3 320 were accurate, but the number of parameters was 19.1M and 24.4M, respectively. In addition, operation speed was 60.9 and 40.1 ms, meaning real-time object detection is impossible. Figure 10 shows the detection results of DRFBNet300 of MS COCO val2017 [23].

**Figure 11:**
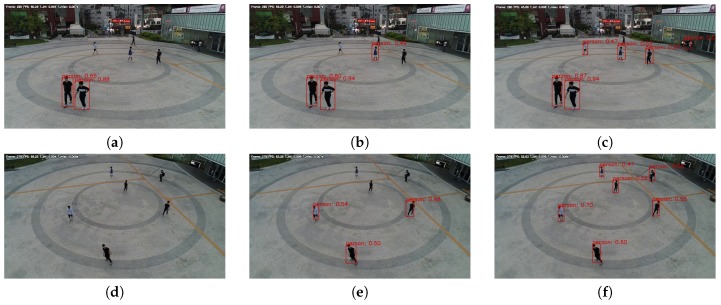
Person detection in bird-view UAV imagery of each model trained on MS COCO. (**a**,**d**) SSD300 results; (**b**,**e**) RFBNet300 results; and (**c**,**f**) DRFNet300 results.

Figure 11 shows the results of person detection in bird-view UAV imagery using MS COCO-trained SSD300, RFBNet300, and our DRFBNet300. Comparing the experiment results of each model, the detection results of DFRBNet300 were better than the other models. This is because the generalization performance of DRFBNet300 is the best and it was designed to detect small objects well.

**Figure 12:**
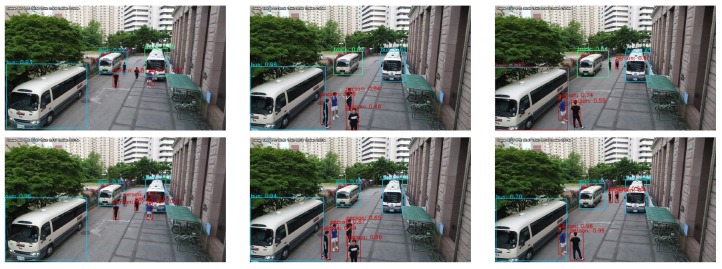
Experiment results of adjacent-frame object-detection (**top**) before and (**bottom**) after applying SIP of MS COCO-trained DRFBNet300.

Figure 12 shows the experiment results of applying the SIP method to DRFBNet300 trained on MS COCO. The model with the SIP method slowed down because the amount of computation increased. However, unlike undetected or misdetected objects when the SIP method is not applied, the accuracy of the applied model was greatly improved.

### 5.2. UAV-Cars Dataset

In this experiment, we trained SSD300, RFBNet300, and our DRFBNet300, which are lightweight single-stage object detectors running in real time, using the training strategies described in Section 4.3. A training set consisting of 4407 images, including 18,945 objects, in the UAV-cars dataset was used for each model’s training. The trained models were evaluated using the UAV-cars validation set consisting of 628 images containing 2637 objects. The validation set was divided into five cases at altitude intervals of 10 m from 10 to 50 m. The True Positive criterion was set to a 0.5 Intersection over Union (IoU) threshold, which was the same value of the PASCAL VOC [24].

**Figure 13:**
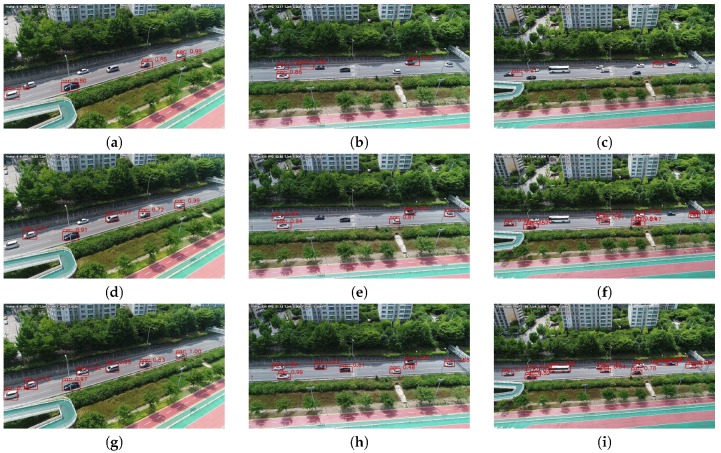
Experiment results of the UAV-cars validation set without applying SIP method. (left to right) Altitudes of 30, 40, and 50 m. Results of (**a**–**c**) SSD300; (**d**–**f**) RFB300; and (**g**–**i**) our DRFBNet300.

Table 4 and Figure 13 show the AP and detection results of models trained on the UAV-cars training set. In Table 4, we can see that DRFBNet300 achieved the highest AP score at all altitudes except for at 10 m. In addition, since inference time was only 17.5 ms, real-time vehicle detection was possible at 57 FPS. In Figure 13, we can see that DRFBNet300 detected small-sized vehicles better than the other models.

In Table 4, accuracy at all altitudes except for at 10 m was greatly improved when the SIP method was applied. Especially as altitude increased, the AP score also further increased. Even at 50 m altitude, the AP score of DRFBNet300 with the SIP method was 57.28, which is a 30.07 AP increase at 27.21 AP when not applied. This is more than double the AP score when it was not applied.

**Figure 14:**
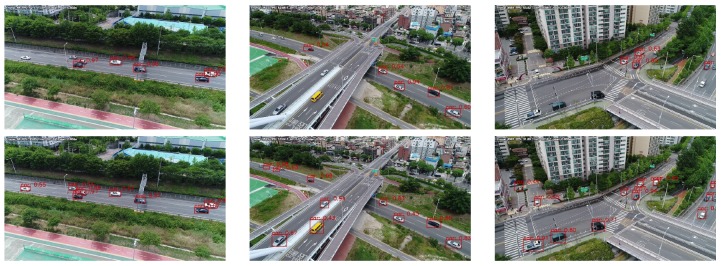
Experiment results of DRFBNet300 trained on UAV-cars dataset (**top**) before and (**bottom**) after applying SIP.

Figure 14 shows UAV imagery vehicle-detection results in the practical case of DRFBNet300 according to whether SIP was applied or not. Figure 14 shows DRFBNet300 with SIP detected vehicles that cannot be detected by normal DRFBNet300 in bird-view UAV imagery at high altitudes. In addition, it ran in real time at 33 FPS even when SIP was applied.

## 6. Conclusions

In this paper, we proposed the use of DRFBNet300 with a DRFB module for bird-view UAV imagery vehicle detection, the UAV-cars dataset to train DRFBNet300, and the SIP method to improve the accuracy of our vehicle detector. The single-stage object-detection model, SSD, has low computational complexity and is fast, but does not detect small objects well. Accuracy is also lower when using a lightweight backbone network for speeding up. DRFBNet300 is a DRFB and RFB basic module attached to the MobileNet v1 backbone SSD300, which is a lightweight object-detection model. The DRFB module was designed to have a multisized RF branch, and dilated convolution was implemented to minimize the increase of computation amount. The multibranched and cascaded structure of our DRFB module improved the quality of feature maps, which improved the accuracy of the vehicle-detection model. We also proposed a UAV-cars dataset consisting of 5035 images containing 21,582 objects, including distortion peculiarities of vehicles in bird-view UAV imagery. Lastly, we proposed the SIP method to improve DRFBNet300 accuracy. DRFBNet300 with the DRFB module achieved the highest score among other lightweight single-stage methods running in real time with 21 mAP at 45 FPS on the MS COCO metric. In the experiment on the UAV-cars dataset, DRFBNet300 also obtained the highest AP score, regardless of whether the SIP method was applied or not at 20–50 m altitudes. The DRFBNet300 increased the accuracy improvement by the SIP method as the UAV altitude increased, and accuracy was improved by more than two times at an altitude of 50 m. Because of DRFBNet300 and the SIP method, the proposed method can more accurately detect vehicles in real-time in UAV imagery at 33 FPS.

## Figures and Tables

**Table 1:** Input and output channels before the cascaded structure of the DRFB module.

Branch 0	Branch 1	Branch 2	Branch 3
Input (19×19×512)			
512/128, BN, ReLU	512/128, BN, ReLU	512/128, BN, ReLU	512/64, BN, ReLU
128/128, BN, ReLU	128/128, BN, ReLU	128/128, BN, ReLU	64/64, BN, ReLU
128/128, -, ReLU	128/128, BN, ReLU	128/128, BN, ReLU	64/96, BN, ReLU
-	128/128, BN, ReLU	128/128, BN, ReLU	96/128, BN, ReLU
-	128/128, -, ReLU	128/128, -, ReLU	128/128, BN, ReLU
-	-	-	128/128, -, ReLU
Concatnation + Conv (BN, 19×19×512)			

**Table 2:** Server PC specification table.

CPU	Inter Core I7-7700K
**RAM**	DDR4 16GB
**GPU**	Nvidia GeForce GTX Titan X (Maxwell)
**O/S**	Ubuntu 16.04 LTS
**GPU Library**	CUDA 9.0 with cuDNN v7.5
**Toolkit**	Pytorch-GPU 1.0.1

**Table 3:** Experiment results of the MS COCO dataset.

Method	Backbone	# of Params	Time (ms)	Avg. Precision, IoU			Avg. Precision, Area		
				0.5:0.95	0.5	0.75	Small	Medium	Large
SSD300		7.8 M	19.8	0.181	0.318	0.181	0.014	0.173	0.369
FSSD300		19.1 M	60.9	0.229	0.402	0.236	0.055	0.258	0.386
YOLOv3 320	MobileNet v1	24.4 M	40.1	0.236	0.407	0.241	0.082	0.240	0.386
RFBNet300		6.8 M	21.3	0.206	0.358	0.209	0.018	0.210	0.381
**DRFBNet300**		7.6 M	22.3	0.210	0.368	0.212	0.018	0.212	0.387

**Table 4:** Experiment results of UAV-cars dataset.

Method	Meta Architecture	Backbone	Time (ms)	AP by Altitude (%)				
				10 m	20 m	30 m	40 m	50 m
W/O SIP method	SSD300	MobileNet v1	13.3	98.59	72.19	36.07	26.73	5.01
	RFBNet300		14.9	99.98	82.81	64.35	54.75	24.35
	DRFBNet300		17.5	99.54	90.19	71.38	55.22	27.21
W/ SIP method	SSD300		21.6	95.58	81.99	58.65	45.54	18.23
	RFBNet300		26.2	94.28	84.73	71.11	64.65	47.09
	DRFBNet300		30.2	94.82	91.13	76.85	68.44	57.28
